# Recent Advances in Conotoxin Classification by Using Machine Learning Methods

**DOI:** 10.3390/molecules22071057

**Published:** 2017-06-25

**Authors:** Fu-Ying Dao, Hui Yang, Zhen-Dong Su, Wuritu Yang, Yun Wu, Hui Ding, Wei Chen, Hua Tang, Hao Lin

**Affiliations:** 1 Key Laboratory for Neuro-Information of Ministry of Education, School of Life Science and Technology, Center for Informational Biology, University of Electronic Science and Technology of China, Chengdu 610054, China; koyee_d@sina.com (F.-Y.D.); huiyang0325@163.com (H.Y.); zhendong__su@163.com (Z.-D.S.); wyang@imu.edu.cn (W.Y.); hding@uestc.edu.cn (H.D.); 2 Development and Planning Department, Inner Mongolia University, Hohhot 010021, China; 3 College of Computer and Information Engineering, Xiamen University of Technology, Xiamen 361024, China; ywu@xmut.edu.cn; 4 Department of Physics, School of Sciences, and Center for Genomics and Computational Biology, North China University of Science and Technology, Tangshan 063000, China; 5 Department of Pathophysiology, Southwest Medical University, Luzhou 646000, China

**Keywords:** conotoxin, superfamily, ion channel, machine learning method

## Abstract

Conotoxins are disulfide-rich small peptides, which are invaluable peptides that target ion channel and neuronal receptors. Conotoxins have been demonstrated as potent pharmaceuticals in the treatment of a series of diseases, such as Alzheimer’s disease, Parkinson’s disease, and epilepsy. In addition, conotoxins are also ideal molecular templates for the development of new drug lead compounds and play important roles in neurobiological research as well. Thus, the accurate identification of conotoxin types will provide key clues for the biological research and clinical medicine. Generally, conotoxin types are confirmed when their sequence, structure, and function are experimentally validated. However, it is time-consuming and costly to acquire the structure and function information by using biochemical experiments. Therefore, it is important to develop computational tools for efficiently and effectively recognizing conotoxin types based on sequence information. In this work, we reviewed the current progress in computational identification of conotoxins in the following aspects: (i) construction of benchmark dataset; (ii) strategies for extracting sequence features; (iii) feature selection techniques; (iv) machine learning methods for classifying conotoxins; (v) the results obtained by these methods and the published tools; and (vi) future perspectives on conotoxin classification. The paper provides the basis for in-depth study of conotoxins and drug therapy research.

## 1. Introduction

Conotoxins are the group of cysteine-rich neurotoxic peptides isolated from the venom of the marine snails of the genus *Conus*. [1]. Mature conotoxins consist of 10 to 30 residues with ≥1 disulfide bonds. By binding to various ion channels, conotoxins possess important biological functions [2]. Conotoxins play key roles in pharmacology and neuroscience as well as new drug development; and have attracted the attention of scientists worldwide [3,4,5,6,7,8,9]. Wang et al. [10] found there was apparent synergistic analgesic effects that were produced by ω-conotoxin MVIIA and morphine in rats. Conantokin-R [11] is a highly potent anticonvulsant with a protective index of 17.5 when tested on an audiogenic mouse model of epilepsy.

Over the last few decades, conotoxins have been the subject of pharmacological interest [12], and have been used in the treatment of various diseases such as Alzheimer’s disease, Parkinson’s disease, epilepsy, chronic pain, and cardiovascular diseases. Conical spirodotoxin, as a non-addictive analgesic, has good prospects. Under the same dose, the effect of conical spirodotoxin is 1000 times higher than that of morphine. Conotoxins have also been characterized by various rapeutic potentials in pre-clinical or clinical trials, such as antinociceptive [13], antiepileptic [14], neuroprotective, and cardioprotective activities [15]. In addition, they also have the potential to cultivate insect-resistant crop varieties and be the candidate of polypeptide pesticide [16,17]. The therapeutic potential of conotoxin is ascribed to their special ion channel-targets in the nervous systems [4]. Thus, they have been regarded as excellent pharmacological probes and potential candidate compounds for drug design for neurological disorders [18].

**Figure 1:**
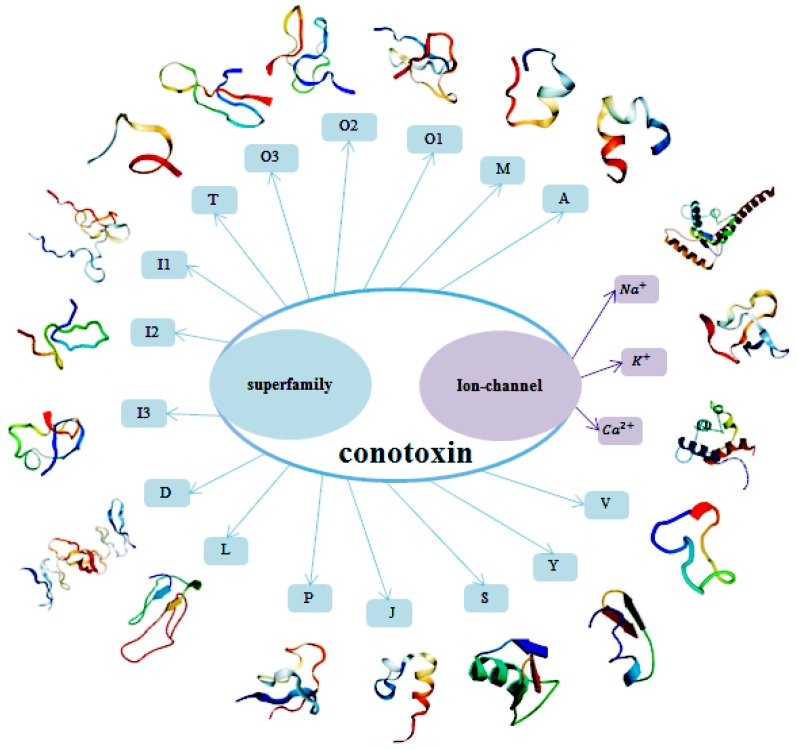
A structural schematic illustration to show the classification of conotoxins in superfamily and ion channel-target. Sixteen major conotoxin superfamilies are A, D, I1, I2, I3, J, L, M, O1, O2, O3, P, S, T, V, and Y. They are also categorized into calcium channel-targeted, sodium channel-targeted, and potassium channel-targeted conotoxins according to their functions.

Based on the N-terminal precursor sequence and disulfide connectivity, uncharted conotoxins may be classified into several superfamilies [19,20]. Currently, conotoxins can be classified into 16 major superfamilies: A, D, I1, I2, I3, J, L, M, O1, O2, O3, P, S, T, V, and Y [4,19,20,21,22,23,24,25]. Each superfamily can be further classified into several families based on the cysteine arrangement. For example, A-superfamily conotoxins are classified into α, αA, and κA families; M-superfamily [26,27] includes μ and ψ families; O-superfamily includes δ, μO, ω, κ, and γ families [22,28]. Due to the high specificity and affinity towards ion channels, conotoxins can also be categorized into calcium channel-targeted conotoxins (Ca-conotoxins), sodium channel-targeted conotoxins (Na-conotoxins), and potassium channel-targeted conotoxins (K-conotoxins) [29]. We draw a structural schematic illustration to show conotoxins classifications of superfamily and ion channel-target (Figure 1).

There are over 100,000 conotoxins in approximately 700 species of cone snails [2]. However, only 8344 conotoxins have been deposited in the Universal Protein Resource (UniProt, 15 May 2017). The functions of most conotoxins are still unknown. With more and more conotoxins being sequenced, determining the function of conotoxins with biochemical experiment-based methods is becoming more and more difficult because of the high cost and long period of wet experiments. Computational methods have provided opportunities to rapidly and accurately identify the categories of conotoxins and know about some functions of conotoxins while avoiding the disadvantages of biochemical experiments-based methods [30,31].

**Figure 2:**
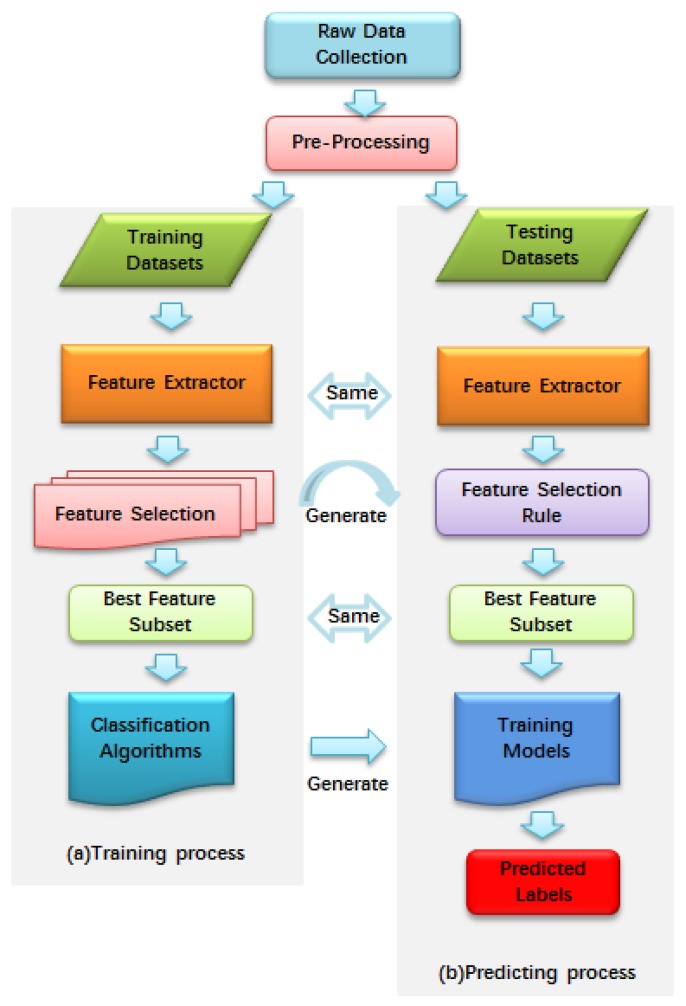
The process framework of conotoxin classification with machine learning methods.

Machine learning approaches have been widely applied in protein or peptide classification by using amino acid composition, *n*-mer amino acid composition, pseudo amino acid composition, position-specific scoring matrix (PSSM) and so on [32,33,34,35,36,37,38,39]. A process framework of protein or peptide classification with a machine learning approach was shown in Figure 2. These methods were also proposed to identify conotoxins superfamily type. A multi-class support vector machine (SVM) was proposed to predict conotoxin superfamily by using pseudo amino acid composition (PseAAC) [24]. Subsequently, Lin et al. improved the accuracy of classifying conotoxin superfamily by using the modified Mahalanobis discriminant [32]. Inspired by these works, Fan et al. proposed a late-model approach and established a webserver called PredCSF for conotoxin superfamily prediction [33]. Zaki et al. used local alignment partition functions to predict conotoxin superfamilies [34]. Then, they introduced a novel method called Toxin-AAM for classifying conotoxin superfamilies [35]. Yin et al. predicted conotoxin superfamilies by using diffusion maps-based feature selection technique [36]. Laht et al. classified conotoxin superfamilies and families based on profile Hidden Markov Models (pHMMs) [37]. Koua et al. established pHMMs for each of the 48 alignments with the hmmbuild script in the HMMER 3.0 package and built a webserver called ConoDictor based on the method [38]. Moreover, they defined 50 position-specific scoring matrices (PSSMs) and 47 hidden Markov models to improve accuracy for conotoxin superfamily prediction [39].

Although these methods and results exemplified above can provide some clues for the study of conotoxins, they only indirectly offer possible function information of conotoxins and they cannot predict the receptor types of the conotoxins. For example, both Delta-conotoxin-like Ac6.1 and Omega-conotoxin-like Ai6.2 belong to the O1 superfamily, but they target different ion channels. The Delta-conotoxin-like Ac6.1 binds to voltage-gated sodium channels, whereas the Omega-conotoxin-like Ai6.2 blocks voltage-gated calcium channels [40]. Thus, it is necessary to develop new computational tools that can recognize the types of ion channel-targeted conotoxins. For the first time, Yuan et al. developed a feature selection technique based on binomial distribution to predict the types of ion channel-targeted conotoxins by using a radial basis function network [41]. Subsequently, they developed a predictor (*iCTX*-*Type*) to improve prediction accuracies [42]. Zhang et al. applied a hybrid feature in the prediction issue [43]. Wu et al. incorporated new properties of residues into PseAAC to predict the types of conotoxins [44]. Recently, Wang et al. combined the analysis of variance and correlation (AVC) with SVM to reduce redundancy of attributes and improve the prediction accuracy and computation speed [45].

In this review, we summarized recent advances in conotoxin classification by using machine learning methods in the following aspects: (i) benchmark dataset construction; (ii) feature extraction method; (iii) feature selection technique; (iv) classification algorithms; (v) prediction accuracy and web servers establishment; and (vi) prospect of conotoxin prediction with machine learning methods.

## 2. Benchmark Datasets

### 2.1. Published Database Resources

Constructing a high quality and reliable benchmark dataset is critical for the protein attribute predictor. Both general databases and special databases play a key role in the construction of bioinformatics benchmark [46,47,48,49]. The general databases include the protein knowledgebase (UniProtKB: http://www.uniprot.org) [50], the protein structure data bank (PDB: http://www.rcsb.org/pdb/home/home.do) [51], and the protein database provided by the National Center for Biotechnology information (NCBI) [52]. Researchers used to collect the data from these molecular biology databases.

For the convenience of users, some special databases were constructed. Here, we mainly introduced the ConoServer (http://www.conoserver.org/), which was a specific database for conotoxins [53,54]. The database collected various kinds of information of conotoxins from SwissProt, GenBank, Protein Data Bank and literatures, including peptide sequences, chemical modifications, and their ability to block the ion channels. At present, the ConoServer has managed 2838 nucleic sequences (from 83 *Conus* species), 6255 protein sequences (from 109 *Conus* species) and 176 3D structures (from 35 *Conus* species) until 16 April 2017, provides a convenient overview of current knowledge on conopeptides and furnishes sequence/structure/activity relationships information, which is of particular interest for drug design research.

### 2.2. Benchmark Dataset Construction

Although the ConoServer contains much information, for the purpose of conotoxin prediction, it is necessary to construct a new benchmark dataset that can be handled by machine learning methods. Generally, a high quality benchmark dataset is constructed in the four following steps. In step 1, samples of conotoxin peptide are acquired from a database with some relevant key words. In step 2, only those proteins with clear functional annotations based on experimental evidence are included. In step 3, the proteins with the annotation information of “immature”, “invalid”, and “fragment” are excluded. In step 4, redundancy and homology bias are reduced by using the program CD-HIT [55] which has been widely used for clustering and comparing protein or nucleotide sequences.

Based on the strict steps above, some high-quality datasets have been constructed for conotoxin superfamilies. Some superfamilies with relatively less members were not considered in some studies [24,32]. The first benchmark dataset of superfamily was called S1, which included 116 mature conotoxin sequences including A (25 entries), M (13 entries), O (61 entries) and T (17 entries) superfamilies [24]. At the same time, they also built a negative dataset containing 60 short peptide sequences that did not belong to any of the four superfamilies (A, M, O or T). The second benchmark dataset S2 contains 261 entries consisting of four superfamilies: A (63 samples), M (48 samples), O (95 samples) and T (55 samples) obtained from the SwissProt [33]. In addition, Lath et al. collected 964 sequences from ConoServer [37]. Koua et al. also acquired 933 samples and 967 samples from Conoserver [38,39].

The benchmark dataset of ion channel-targeted conotoxins was also constructed based on the Uniprot. The function type of conotoxins was obtained by searching Gene Ontology. The first benchmark dataset I1 established by Yuan et al. included 112 sequences (24 K-conotoxins, 43 Na-conotoxins, and 45 Ca-conotoxins) [41]. Ding et al. [42], Wu et al. [44] and Wang et al. [45] also established their models based on this dataset. In addition, Zhang et al. built a new dataset called I2 containing 145 samples (26 K-conotoxins, 49 Na-conotoxins and 70 Ca-conotoxins) [43]. The benchmark datasets are provided in Table 1.

## 3. Conotoxin Sample Description Methods

In the process of protein classification with machine learning methods, the second step is to represent protein samples. Two strategies may be adopted: the continuous model and the discrete model. In the continuous model, the BLAST or FASTA programs are used to search homology. For a highly similar sequence (sequence identity ≥40%) in the searching dataset, its predictive results are always good. Thus, the similarity-based method is straightforward and intuitive. However, if a query protein has no similar sequence in the training dataset, these methods cannot work. Therefore, various discrete models were recommended [24,32,33,34,35,36,41,42,43,44,45,56]. The way to formulate conotoxin samples with discrete models is provided below.

### 3.1. Amino Acid Compositions and Dipeptide Compositions

The amino acid compositions (AAC) and dipeptide compositions are the most widely used features to formulate the protein samples, and can be formulated as:

(1) $$X_{20}={\left[x_{1}\cdots x_{i}\cdots x_{20}\right]}^{T},$$

(2) $$Y_{400}={\left[y_{1}\cdots y_{i}\cdots y_{400}\right]}^{T},$$

where $x_{i}$ (*i* = 1,2,... , 20) and $y_{i}$ (*i* = 1, 2,..., 400) are, respectively, the absolute occurrence frequencies of 20 native amino acids and 400 dipeptides, which, respectively, describe the sequence composition and neighborhood information of residues.

Based on the two kinds of parameters above, Lin et al. [32] developed a method to predict conotoxin superfamilies by combining the increment of diversity with modified Mahalanobis discriminant. Recently, the 400 dipeptide compositions were also used to represent a conotoxin sequence by Wang et al. [45].

### 3.2. Pseudo Amino Acid Composition

The pseudo amino acid composition (PseAAC) is a widely used strategy for peptide sample description in protein classification [57,58]. PseAAC can not only include amino acid composition, but also the correlation of physicochemical properties between two residues [59]. Its merits have been demonstrated in a series of studies [24,44,57,58].

Mondal et al. constructed a model by using Type-I PseAAC to formulate samples for predicting superfamilies of conotoxins [24]. The Type-I PseAAC is also called parallel correlation PseAAC, which contains 20 + *λ* components. The number ‘20’ reflects the occurrence frequency of one of the 20 native amino acids in a protein P and λ reflects the rank of correlation and is a non-negative integer. In the discrete descriptor, an arbitrary conotoxin (*P*) can be expressed by a 20 + *λ*-dimensional vector and is defined as follows:

(3) $$P={\left[x_{1}\cdots x_{20}x_{20+1}\cdots x_{20+\lambda }\right]}^{T},$$

where

(4) $$x_{u}=\{\begin{array}{c} \frac{f_{u}}{\sum_{i=1}^{20}f_{i}+\omega \sum_{j=1}^{\lambda }\theta_{j}},\;\left(1\leq u\leq 20\right)\\ \frac{\omega \theta_{u-20}}{\sum_{i=1}^{20}f_{i}+\omega \sum_{j=1}^{\lambda }\theta_{j}},\left(20+1\leq u\leq 20+\lambda \right) \end{array},$$

where $f_{i}$ is denoted as the normalized frequency of the 20 residues in a conotoxin. ω is weight factor for sequence order effect and was previously defined as 0.7 [24]. $\theta_{j}$ is the *j*-tier sequence correlation factor and calculated as:

(5) $$\theta_{j}=\frac{1}{L-j}\sum_{i=1}^{L-j}Ѳ\left(R_{i},R_{i+1}\right),\;(j<L),$$

where $\theta_{j}$ is the *j*-th tire correlation factor that reflects the sequence order correlation between all the *j*-th most contiguous residues along a protein sequence. In addition, the correlation function is given by:

(6) $$Ѳ\left(R_{i},R_{j}\right)=\frac{1}{k}\left\{{\left[H_{1}\left(R_{j}\right)-H_{1}R_{i}\right]}^{2}+{\left[H_{2}\left(R_{j}\right)-H_{2}R_{i}\right]}^{2}+\cdots +{\left[H_{k}\left(R_{j}\right)-H_{k}R_{i}\right]}^{2}\right\},$$

where *k* is the number of factors and $H_{l}\left(R_{i}\right)$ is the *l*-th physiochemical properties of the residue $R_{i}$:

(7) $$H_{l}\left(R_{i}\right)=\frac{H_{l}^{0}\left(i\right)-\sum_{i=1}^{20}\left(H_{l}^{0}\left(i\right)/20\right)}{\sqrt{\frac{\sum_{i=1}^{20}{\left[H_{l}^{0}\left(i\right)-\sum_{i=1}^{20}\left(H_{l}^{0}\left(i\right)/20\right)\right]}^{2}}{20}}},$$

where $H_{l}^{0}\left(i\right)$ is the *l*-th original value of the *i*-th residue. The numerical indices 1, 2, 3,⋯, 20, respectively, represent the 20 native amino acids: A, C, D, E, F, G, H, I, K, L, M, N, P, Q, R, S, T, V, W, and Y. The five factors of polarity index, secondary structure factor, molecular size, relative amino acid composition in various proteins and electrostatic charge were used in the model of Mondal et al. [24].

Wu et al. [44] used the Type-II PseAAC, which is also called series correlation PseAAC, to formulate their samples. In the descriptor, an arbitrary conotoxin (*P*) is expressed as a vector containing ($20^{2}\;$+ 3*λ*) components:

(8) $$P={\left[x_{1}\cdots x_{400}\cdots x_{400+3\lambda }\right]}^{T},$$

where $x_{1}\cdots x_{400}$ denote the frequencies of $20^{2}$ dipeptides. The ‘3’ is the number of amino acid properties, namely, rigidity, flexibility, and irreplaceability; *λ* reflects the rank of correlation, which is the same as that in Type-I PseAAC:

(9) $$x_{u}=\{\begin{array}{c} \frac{f_{u}}{\sum_{i=1}^{400}f_{u}+\omega \sum_{j=1}^{3\lambda }\tau_{j}},\;\left(1\leq u\leq 400\right)\\ \frac{\omega \tau_{u}}{\sum_{i=1}^{400}f_{u}+\omega \sum_{j=1}^{3\lambda }\tau_{j}},\;\left(400+1\leq u\leq 400+3\lambda \right) \end{array},$$

where *ω* is weight factor for sequence order effect; and $f_{u}$ was the normalized frequency of the 400 dipeptides in conotoxin (*P*);

(10) $$f_{u}=\frac{n_{u}}{\sum_{u}n_{u}},$$

where $n_{u}$ denotes the number of occurrences of *𝑢*-th dipeptide in conotoxin (*P*); $\tau_{u}$ in Equation (9) is the correlation factor of the physicochemical properties between residues:

(11) $$\{\begin{array}{c} \tau_{1}=\frac{1}{L-1}\sum_{K=1}^{L-1}H_{k,k+1}^{1}\\ \tau_{2}=\frac{1}{L-1}\sum_{K=1}^{L-1}H_{k,k+1}^{2}\\ \vdots \\ \tau_{n}=\frac{1}{L-1}\sum_{K=1}^{L-1}H_{k,k+1}^{n}\\ \tau_{n+1}=\frac{1}{L-2}\sum_{K=1}^{L-2}H_{k,k+2}^{1}\\ \tau_{n+2}=\frac{1}{L-2}\sum_{K=1}^{L-2}H_{k,k+2}^{2}\\ \vdots \\ \tau_{n+n}=\frac{1}{L-2}\sum_{K=1}^{L-2}H_{k,k+2}^{n}\\ \vdots \\ \tau_{n\lambda }=\frac{1}{L-\lambda }\sum_{K=1}^{L-\lambda }H_{k,k+\lambda }^{n} \end{array}\;\left(\lambda <L\right),$$

where $H_{k,k+\lambda }^{n}$ is the correlation function:

(12) $$H_{k,k+\lambda }^{n}=h^{n}\left(R_{k}\right)\cdot h^{n}\left(R_{k+\lambda }\right),$$

where $h^{n}\left(R_{k}\right)$ is the *n*-th kind of the physicochemical values of the residue $R_{k}$. The values should be converted to standard type:

(13) $$h^{n}\left(R_{k}\right)=\frac{h_{0}^{n}\left(R_{k}\right)-\langle h_{0}^{n}\left(R_{k}\right)\rangle }{SD\langle h_{0}^{n}\left(R_{k}\right)\rangle },$$

where $h_{0}^{n}\left(R_{k}\right)$ is the original physicochemical value of the *k*-th residue.

Both Type-I and Type-II PseAAC can not only describe the information of the constituent elements of the conotoxin sequence, but also reflect the long-range correlation information of residues’ physicochemical properties. Therefore, PseAAC can usually produce better prediction accuracy compared with the traditional peptide frequency. Because Type-II PseAAC considers the contributions of each kind of physicochemical property, it exhibits a better prediction performance as shown in Ref. [44]

### 3.3. Hybrid Features

Instead of using a single discrete model, different features were used to describe conotoxin samples. Recently, the 246 physicochemical properties of residues obtained from APDbase [60] were used to formulate protein samples [33,36]:

(14) $$\{\begin{array}{c} P_{1}=R_{1}^{1}R_{2}^{1}R_{3}^{1}R_{4}^{1}\cdots R_{L}^{1}\\ P_{2}=R_{1}^{2}R_{2}^{2}R_{3}^{2}R_{4}^{2}\cdots R_{L}^{2}\\ \vdots \\ P_{246}=R_{1}^{246}R_{2}^{246}R_{3}^{246}R_{4}^{246}\cdots R_{L}^{246} \end{array}.$$

By using the maximal overlap discrete wavelet transform (MODWT) to construct the eigenvectors [61], a conotoxin sample can thus be represented by a 1230-dimensional feature vector ((1 + 1 + 3) × 246 = 1230):

(15) $$F_{MODWT}=\left[f_{1}^{1,1},f_{2}^{1,2},f_{3}^{1,3},f_{4}^{1,4},f_{5}^{1,5},f_{6}^{2,1},f_{7}^{2,2},f_{8}^{2,3},f_{9}^{2,4},f_{10}^{2,5},\cdots f_{1230}^{246,5},\right].$$

In addition, three characteristics were also incorporated in their model: 20D features of evolutionary information, 3D secondary structural (SS) information, and 20D AAC. Therefore, the final feature set to formulate conotoxin sample was a (1230 + 20 + 3 + 20)1273D vector.

Compared with the above two methods, the method combines with several models to represent protein samples. Thus, the bias caused by a single discrete model can be significantly reduced.

## 4. Feature Selection Techniques

Feature selection is important in pattern recognition for the insight gained from determining relevant modeling variables. By feature selection, generalization ability of prediction model will improve, information redundancy or noise will be excluded; and the dimension disaster will be resolved [62]. It can significantly increase the comprehensibility of classifier models and often build a better model [63]. The ultimate goal of feature selection is to find the best feature subset that can produce the maximum accuracy and to establish a robust prediction model. Currently, many feature selection techniques have been developed to optimize a feature set, such as principal component analysis (PCA) [64], minimal-redundancy-maximal-relevance (mRMR) [65], maximum-relevancy-maximum-distance (MRMD) [66], diffusion maps [36] and the analysis of variance (ANOVA) [67]. The following feature selection techniques have been used in conotoxin prediction.

### 4.1. Binomial Distribution

Binomial distribution is a discrete probability and can deal with the experiments that have two types of results. Thus, Yuan et al. [41] proposed using the binomial distribution to perform feature selection in order to improve the accuracy of conotoxin prediction. In their model, the confidence level (*CL*) of each feature was calculated by:

(16) $$CL_{ij}=1-\sum_{n=n_{ij}}^{N_{i}}\frac{N_{i}!}{n!\left(N_{i}-n\right)!}p_{j}^{n}{\left(1-p_{j}\right)}^{N_{i}-n},$$

where $CL_{ij}$ is the confidence level of the *i*-th dipeptide in the *j*-th type; $N_{i}$ represents the total number of the *i*-th dipeptide in the dataset; $n_{ij}$ represents the occurrence number of the *i*-th dipeptide in the *j*-th type and the sum is taken from $n_{ij}$ to $N_{i}$; the probability $p_{j}$ is the relative frequency of Type *j* in the database; the confidence level of peptide *i* in benchmark dataset is defined as follows:

(17) $$CL_{i}=max\left\{CL_{i\;k},CL_{i\;Na},CL_{i\;Ca}\right\},$$

All features can be ranked in descending order according to their *CL*s. According to the principle of feature selection, the $CL_{i}$ reveals the degree that the *i*-th feature is related to the group variables. The larger *CL* the feature is, the higher its contribution to the classification. The binomial distribution-based technique is a powerful statistical method that can extract the over-represented motifs; however, it needs more computational resources.

### 4.2. Relief Algorithm

Zhang et al. [43] proposed another feature selection technique called relief algorithm in conotoxin classification. The relevance between the features and class labels can be depicted by this algorithm [68]. Based on the ability of the feature to distinguish the near samples, the weighted features can be formulated by [69]:

(18) $$W_{P}^{i+1}=W_{P}^{i}-\frac{diff\left(Y,x_{i},H\left(x_{i}\right)\right)}{m}+\frac{diff\left(S,x_{i},M\left(x_{i}\right)\right)}{m},$$

(19) $$diff\left(*,x,y\right)=\{\begin{array}{c} \|x-y\|,x\neq y,\\ 0,\;x\neq y, \end{array}$$

where $W_{P}^{i}$ and $W_{P}^{i+1}$ denote the current and next weighting values, respectively. *p* stands for a given feature; $x_{i}$ denotes the *i*-th sample sequence; $H\left(x_{i}\right)$ represents the nearest neighbor samples from the same class label against $x_{i}$; $M\left(x_{i}\right)$ represents the nearest neighbor samples from the different class labels against $x_{i}$; *Y* and *S* are, respectively, the sample sets with the same and different class labels against $x_{i}$; *m* denotes the number of random samples; the function of diff(*,x,y) is used to calculate the distance between the random samples.

The algorithm is not dependent on heuristics, runs in low-order polynomial time, and is noise-tolerant and robust to feature interactions; however, it does not discriminate between redundant features.

### 4.3. F-Score Algorithm

Ding et al. [42] and Wu et al. [44] used the *F*-score to sort the features for conotoxin classification:

(20) $$F\left(i\right)=\frac{\sum_{k=1}^{3}{\left({\bar{x}}_{i}^{k}-{\bar{x}}_{i}\right)}^{2}}{\sum_{k=1}^{3}\left(1/\left(N_{k}-1\right)\right)\sum_{j=1}^{N_{k}}{\left(x_{ij}^{k}-{\bar{x}}_{i}^{k}\right)}^{2}},$$

where ${\bar{x}}_{i}^{k}$ is the average frequency of the *i*-th feature in the *k*-th dataset; ${\bar{x}}_{i}$ the average frequency of the *i*-th feature in all of the datasets concerned;$\;x_{ij}^{k}$ is the frequency of the *i*-th feature of the *j*-th sequence in the *k*-th dataset; $N_{k}$ is the number of peptide samples in the *k*-th dataset. The larger the *F* value is, the better the predictive capability the feature has. A python script fselect.py downloaded from https://www.csie.ntu.edu.tw/~cjlin/libsvmtools/ can be used to perform the *F*-score calculation and rank the features.

The *F*-score is a simple but effective technique for evaluating the discriminative power of each feature in the feature set. It has strict mathematical definition but does not take the true negatives into account.

### 4.4. Diffusion Map Reduction

For conotoxin superfamily classification, Yin et al. [36] proposed using the diffusion maps to project the data into diffusion space. Diffusion maps algorithm can effectively reduce the data dimensionality while keeping the original data structure [70]. Thus, high-dimensional data can be projected into a low-dimensional space based on diffusion maps, while the intrinsic properties are kept almost invariant. Compared with other methods, the diffusion maps algorithm is robust to noise perturbation and is computationally inexpensive. It has strict mathematical definition but does not take the true negatives into account [71].

It is assumed that there is a dataset Ω with *N* observations and each of them has *p* attributes. If a weighted graph on the dataset is defined, the margin between two observations *x* and *y* is defined as:

(21) $$w\left(x,y\right)=exp\left(-\frac{{\left(x-y\right)}^{2}}{\varepsilon }\right),$$

where ${\left(x-y\right)}^{2}$ is the application dependent dissimilarity between *x* and *y*; the degree of node *x* is defined as:

(22) $$d\left(x\right)=\sum_{z\in \Omega }w\left(x,z\right).$$

Next, a Markov random walk over the weighted graph can be constructed. The transition probability from *x* to *y* in one-step is:

(23) $$p\left(x,y\right)=\frac{w\left(x,z\right)}{d\left(x\right)},$$

where w(x,z) and d(x) are, respectively, defined in Equations (21) and (22).

Then, a transition matrix R of size N × N can be built, and each element of R is calculated by Equation (23). R is the transition matrix for a Markov random walk and can be used to calculate the transition probability matrix $R_{t}$, where each entry in $R_{t}$ represents the probability going from *x* to *y* in *t* steps. Based on $R_{t}$, the stationary distribution of the random walk $\varphi_{0}\left(x\right)$ can be calculated:

(24) $$\varphi_{0}\left(x\right)=\frac{d\left(x\right)}{\sum_{z\in \Omega }d\left(z\right)}.$$

The next step is to define the diffusion distance between two points at the scale *t* as:

(25) $$D_{t}^{2}\left(x,y\right)=\sum_{z}\frac{{\left(R_{t}\left(x,z\right)-R_{t}\left(y,z\right)\right)}^{2}}{\varphi_{0}\left(z\right)}.$$

The diffusion map at scale *t* can project the data *x* from the original space into the *m*-dimensional diffusion space by taking the first *m* eigenvectors as follows:

(26) $$x\to \left[\frac{\lambda_{1}}{1-\lambda_{1}}\psi_{1}\left(x\right),\frac{\lambda_{2}}{1-\lambda_{2}}\psi_{2}\left(x\right),\cdots ,\frac{\lambda_{m}}{1-\lambda_{m}}\psi_{m}\left(x\right)\right],$$

where $\lambda_{j}$ and $\psi_{j}$ are, respectively, the eigenvalue and right eigenvector of $R_{t}$; *m* is the final reduced dimension.

### 4.5. Analysis of Variance

In order to select optimal features from the 400D dipeptide compositions, Wang et al. classified the ion channel-targeted conotoxins with the analysis of variance (ANOVA) method [45]. The variance-based analysis is used to calculate the ratio of the variance among groups and the variance within the group for each attribute [72,73]. It has a good foundation of statistics and can test the feature difference between groups intuitively. The formula expressions are as follows:

(27) $$F\left(u\right)=\frac{S_{b}^{2}\left(u\right)}{S_{w}^{2}\left(u\right)}.$$

The *F* value represents the *u*-th dipeptide, and $S_{b}^{2}\left(u\right)$ is the variance between groups, $S_{w}^{2}\left(u\right)$ is the variance within groups. The calculation methods are shown in Equations (28) and (29), respectively:

(28) $$S_{b}^{2}\left(u\right)=\frac{SS_{b}\left(u\right)}{K-1},$$

(29) $$S_{w}^{2}\left(u\right)=\frac{SS_{w}\left(u\right)}{N-K},$$

where *K* is the total of classes; *N* is the total of samples; $SS_{b}\left(u\right)$ is the sum of the squares between the groups; and $SS_{w}\left(u\right)$ is the sum of squares within the groups.

### 4.6. Feature Selection Process

Picking out informative features can overcome the high-dimensional disaster, reduce information redundancy, exclude noise, and improve the accuracy and robust of the proposed models. Obviously, the most objective and strict method to select the best feature subset is to examine the performance of all the feature combinations. However, the computation time is too long. Taking a 20-dimensional feature vector as an example, there are 1,048,575 possible combinations. Thus, feature selection techniques described above are developed to economize the computational time and source.

The incremental feature selection (IFS) is a popular strategy to determine the optimal feature subset. The selection process is described as follows. At first, all features are ranked according to a score obtained from one of the feature selection techniques described above. Subsequently, the feature subset is built from the first feature in the ranked feature set. Furthermore, a new feature subset is built when the second feature is added. This process is repeated from the first feature to the last feature until all candidate features are added. For each feature subset, the machine learning methods are used to investigate their performance with cross-validation [57,74,75,76,77]. The highest accuracy is produced by the best feature subset, which is selected to build the final prediction model. The machine learning methods in conotoxin prediction is discussed below.

## 5. Prediction Algorithms

The four key steps for conotoxin classification are to select a highly efficient and powerful machine learning method to make a predictive decision. In the prediction, the classification function or classification model was constructed with a machine learning method for predicting the input conotoxin to a given category.

### 5.1. Support Vector Machine

Support Vector Machine (SVM) was originally developed by Vapnik et al. [78]. As SVM is always suitable for small sample, SVM has been widely used to deal with many pattern recognition problems [42,79,80,81,82,83,84,85,86,87], and also some hierarchical classification [88]. As shown in Table 1, the number of train data is from 13 to 95 for each type; thus, several works used SVM to predict conotoxin types [24,32,33,34,35,36,37,38,39,42,43,44,45].

The basic idea of SVM is to transform the input vector into a high-dimensional Hilbert space and seek a separating hyperplane in this space. Gaussian Radial Basis Function (RBF) kernel function ($K_{Gaussion}\left(x_{i},x_{j}\right)=e^{\frac{{\left\|x_{i}-x_{j}\right\|}^{2}}{2\sigma }}$) is a widely used kernel function because of its high performance in non-line classification.

In order to reduce the programming burden of researchers, some software packages including LIBSVM, mySVM and SVMLight [89,90] have been developed and can be freely downloaded from the internet. LIBSVM is the most popular software to implement SVM and can be downloaded from https://www.csie.ntu.edu.tw/~cjlin/libsvm/. A grid search strategy with cross-validation test is always utilized to obtain the best values for the regularization parameter *C* and kernel parameter *g*.

### 5.2. Profile Hidden Markov Models

Profile Hidden Markov Models (pHMMs) are statistical models for capturing position-specific information [91]. The pHMMs provide a formal probabilistic framework for sequence comparison [92] and leverage the information contained in a sequence alignment to improve detection of distantly related sequences [93,94]. More recently, Hidden Markov Models have been extended to pairwise Markov models and triplet Markov models for the consideration of more complex data structures [95] and the model of non-stationary data [96]. Obviously, compared to the classic tool BLAST [97], pHMMs can more accurately detect remote homologs and provide more information by using a statistical representation of a multiple sequence alignment. Recently, a pHMM has been built for each subset using *hmmbuild* from the HMMER package and can be acquired from http://www.hmmer.org/. In addition, more packages about pHMMs can be acquired [91].

### 5.3. K-Local Hyperplane Distance Nearest Neighbor Algorithm

The *K*-local hyperplane distance nearest neighbor algorithm (HKNN) [98] was introduced to overcome the generalization problems of the well-known *K*-nearest neighbor algorithm (KNN). Unlike SVM, it can be used to establish a nonlinear decision surface directly in the original sample space with a local linear manifold. With the HKNN method, the closest *K* neighbors should be firstly found to test the samples for each class. Then, these neighbors are used to build the local linear manifolds of the classes. Finally, the query is allocated to the class that is associated with the closest manifold.

Suppose there are *C* classes in the training set. Let $V_{i}^{k}\left(x_{q}\right)=\left\{x_{1}^{i},x_{2}^{i},\cdots ,x_{k}^{i}\right\}$ represent the set of *K* nearest samples of the tested sample $x_{q}\in {ℵ}^{m}$ in the training set belonging to the *i*-th class. Here, the dimension of the sample space *m* is assumed to be larger than or equal to *K*. The local affine hull of each class is defined in terms of the closest *K* sample vectors as:

(30) $$LH_{i}^{k}\left(x_{q}\right)=\left\{v|v=u_{i}+\sum_{i=1}^{l_{i}}\beta_{j}^{i}z_{j}^{i},\beta_{j}^{i}\in {ℵ}^{m}\right\},\;i=1,\cdots ,C,$$

where $u_{i}=\frac{1}{K}\sum_{j=1}^{K}x_{j}^{i}$, $z_{j}^{i}$ are the linearly independent vectors obtained from the difference vectors $\left\{x_{1}^{i}-u_{i},x_{2}^{i}-u_{i},\cdots ,x_{K}^{i}-u_{i}\right\}$; $l_{i}$ is the number of linearly independent difference vectors and $l_{i}\leq K-1$.

In order to classify a query $x_{q}$, the minimum distances between the query vector and the local manifolds should be computed as follows:

(31) $$\mathrm{dis}\left(x_{q},LH_{i}^{K}\left(x_{q}\right)\right)=\underset{v\in LH_{i}^{K}\left(x_{q}\right)}{\min}\|x_{q}-v\|=\underset{\beta_{i}\in {ℵ}^{l_{i}}}{\min}\|x_{q}-u_{i}-Z_{i}\beta_{i}\|,\;i=1,\cdots ,C.$$

Thus, the query is assigned to the class whose manifold is the closest to $x_{q}$. The details about HKNN can be obtained from the results reported by Yin et al. [36].

### 5.4. Mahalanobis Discriminant

The Mahalanobis distant is a measure of the distance between a point *P* and a distribution *D*, introduced by P. C. Mahalanobis in 1936 [99]. If *P* is at the mean of *D*, this distance is zero and the mean grows as *P* moves away.

Mahalanobis Discriminant has been widely used in cluster analysis and data classification [100]. Due to the imbalance of data samples, based on Bayes theory, the modified Mahalanobis Discriminant was deduced [101].

The modified Mahalanobis Discriminant (*MD*) between test sequence *x* and training set $x_{§}$ can be calculated as:

(32) $$MD_{§}={\left(x-{\bar{x}}_{§}\right)}^{T}\sum_{§}^{-1}\left(x-{\bar{x}}_{§}\right)+\log\left|\sum_{§}\right|,$$

where ${\bar{x}}_{§}$ and $\sum_{§}$ are, respectively, the group mean and covariance matrix for §-th training set; ${\left(x-{\bar{x}}_{§}\right)}^{T}\sum_{§}^{-1}\left(x-{\bar{x}}_{§}\right)$ denotes the square of Mahalanobis distance between test sequence *x* and training set $x_{§}$.

MD is unitless and scale-invariant, and takes into account the correlations of the features. According to the principle of similarity, the smaller the *MD* between test sequence *x* and training set $x_{§}$ is, the higher the probability that sequence *x* belongs to class § is.

### 5.5. Radial Basis Function Network

The RBF network is a special type of Artificial Neural Network (ANN). Due to its faster training procedure and better approximation capabilities, it has been widely used in protein prediction fields [102], and noncoding RNA identification [103]. The RBF network can approximate any nonlinear function with sufficient neurons in the hidden layer. A typical RBF network is composed of three layers: an input layer, a hidden layer and a linear output layer. Training is usually carried out in two stages: (1) fixed width and centers, and (2) fixed weights. This can be demonstrated by considering the different properties of nonlinear hidden neurons versus linear output neurons.

Generally, the RBF network is modeled by the following relation:

(33) $${\hat{y}}_{k}=\sum_{i=1}^{m}\omega_{ik}R_{i}\left(x\right),\;\left(k=1,2,\cdots ,p\right),$$

where $R_{i}\left(x\right)$ represents the RBF and is expressed as:

(34) $$R_{i}\left(x\right)=\exp\left(-\frac{{\left\|x-c_{i}\right\|}^{2}}{2\sigma_{i}^{2}}\right),\;\left(i=1,2,\cdots ,m\right),$$

where $\left\|x-c_{i}\right\|$ represents Euclidean norm; $c_{i}$, $\delta_{i}$, and $R_{i}$ are the center, the width and the output of the *i*-th hidden unit, respectively.

The WEKA [104] soft package is used to execute the RBF network with default parameters.

### 5.6. Random Forest Algorithm

The Random Forest (RF) algorithm is also a popular learning algorithm and has been successfully employed in dealing with various biological prediction problems [105,106,107,108]. The principle of RF is based on the training of multiple decision trees. It just needs two parameters: one is the number of building decision trees *t*, another is the number of input features to be considered when each node of the decision tree splits *m*. By establishing many tree predictors, the type of a new sample can be determined. The results obtained from many experiments have shown that combining multiple trees generated in randomly selected subspaces can significantly improve the prediction accuracy. The algorithm can produce a high accuracy classifier and handle a large number of input variables with fast learning process. For an unbalanced dataset, it can balance the random error. For more detailed information about the RF algorithm, readers can refer to the http://www.stat.berkeley.edu/~breiman/RandomForests/cc_home.htm.

## 6. Prediction Accuracy

In this section, we listed the commonly-used metrics for the performance evaluation of proposed models and introduced the published results.

### 6.1. Commonly-Used Evaluation Metrics

A jackknife test can yield a unique result for a given benchmark dataset and has been wildly applied in various predictions [109,110]. A set of metrics, namely, sensitivity (*Sn*), average accuracy (*AA*) (or called average sensitivity) and overall accuracy (*OA*) are commonly used to quantitatively estimate the accuracy of the models and respectively calculated as:

(35) $$Sn=\frac{TP}{TP+FN},$$

(36) $$AA=\frac{\sum Sn}{\mu },$$

(37) $$OA=\frac{TP}{TP+TN+FP+FN},$$

where *TP*, *FP*, *TN*, and *FN*, respectively, denote the number of true positives, false positives, true negatives, and false negatives; μ is the type of samples.

The receiver operating characteristic (ROC) curve [111] shows the predictive capability. The ROC curve can also present the model behavior of the true positive rate (sensitivity) against the false positive rate (1-specificity) in a visual way. The area under the ROC (auROC) is calculated to quantitatively and objectively measure the performance of the proposed method. A perfect classifier gives auROC = 1 and the random performance gives auROC = 0.5.

### 6.2. Published Results

Based on the benchmark dataset S1, by using Multi-class SVMs combined with PseAAC, Mondal et al. [24] achieved an overall accuracy of 88.1%, which was higher than those obtained by other methods like BLAST and ISort (Intimate Sorting) predictor. In order to improve the accuracy, Lin et al. [32] proposed a new algorithm that combined increment of diversity with modified Mahalanobis discriminant. The algorithm can reduce the dimension of inputting vector, extract important classify information, and improve the calculation efficiency. The average sensitivity and specificity respectively reached 88% and 91% in the jackknife cross-validation test. Zaki et al. developed a scoring system called SVM-Free score based on local alignment partition functions [34] and increased the average sensitivity and specificity to 97.42% and 99.17%, respectively. Based on SVM-Free score method, a soft package was constructed and could be freely downloaded from http://faculty.uaeu.ac.ae/nzaki/SVM-Freescore.htm. Based on this work, a novel method called Toxin-AAM [35] was introduced with evolutionary information and amino acid composition and the average sensitivity reached 94.5% in jackknife cross-validation test.

Based on the benchmark dataset S2, Fan et al. [33] proposed a novel method called *PredCSF* for predicting the conotoxin superfamily by using modified one-versus-rest SVMs. *PredCSF* can realize an overall accuracy of 90.65% in jackknife cross-validation tests. A user-friendly webserver was established and could be freely accessible at http://www.csbio.sjtu.edu.cn/bioinf/PredCSF/. Yin et al. [36] proposed an improved HKNN version called dHKNN algorithm for predicting conotoxin superfamily by considering the local density information in the diffusion space. The overall accuracy of 91.90% was obtained by the jackknife cross-validation test on the benchmark dataset S2. The results indicated that the proposed dHKNN was more promising.

Based on the dataset of ConoServer, Laht et al. [37] acquired 964 sequences and built 62 profile Hidden Markov Models (pHMMs) for the classification of all the described conopeptide superfamilies and families based on the primary sequences. As a result, the mature peptide models realized an accuracy of 96% and the propeptide and signal peptide models got an accuracy of 100%. Koua et al. [38] constructed pHMMs for each of the 48 alignments using the HMMbuild script from the HMMER 3.0 package [112] based on 933 samples of conotoxin superfamily. The model obtained promising discriminative abilities with the sensitivity of ~95% and specificity of ~99%. Based on the model, they published the webserver *ConoDoctor* to predict the conotoxin superfamily, and the package could be freely downloaded from http://conco.ebc.ee. For further improving the accuracy, they established 50 position-specific scoring matrices and 47 hidden Markov models based on 967 sequences from ConoServer [39] and realized the sensitivity of 99.42% and specificity of 92.81%, respectively. Although the accuracies of these models are high, the benchmark datasets used in these models are not objective. Many redundant sequences are included in these datasets. Moreover, the some samples lack biochemical experimental proofs.

Based on the benchmark dataset I1, Yuan et al. [41] predicted the types of ion channel-targeted conotoxins by using binomial distribution and radial basis function network, and achieved an average accuracy of 89.7% and overall accuracy of 85.7% in the prediction of three types of ion channel-targeted conotoxins in the jackknife cross-validation test. The model provides the valuable instructions for theoretical and experimental studies on conotoxins. For further improving the accuracy, Ding et al. [42] used the SVM to classify three kinds of samples based on the feature selection technique *F*-score. The average sensitivity and the overall accuracy respectively reached 90.3% and 91.1%, which are higher than those of the RBF network-based method [41]. For the convenience of the vast majority of experimental scientists, they provided the webserver *iCTX*-*Type*, and user guide details could be obtained from http://lin.uestc.edu.cn/server/iCTX-Type. By incorporating new properties of residues into pseudo amino acid composition, Wu et al. [44] achieved a higher overall accuracy of 94.6%. Recently, an overall accuracy of 91.98% with an average accuracy of 92.17% was obtained by the AVC-SVM model proposed by Wang et al. [45].

Based on the benchmark dataset I2, Zhang et al. [43] proposed a random forest based predictor called ICTCPred for the prediction of the types of ion channel-targeted conotoxins and yielded the satisfactory performance with an average accuracy of 91.0%.

The detailed results obtained by these theoretical methods are provided in Table 2. The published webservers are listed in Table 3. Although many methods have been proposed to predict superfamily types and ion channel-target types of conotoxins, only a few tools were established based on the proposed methods. PsedCSF provides not only a free webserver, but also a stand-alone soft package. The establishment of ConoDictor is based on the cone snail genome project for health. The project website also provides a database called ConoDB, which collects the peptide sequences from cone snails stored in NCBI and Uniprot. The iCTX-Type is the only webserver for the prediction of the types of ion channel-target conotoxins.

## 7. Conclusions

Conotoxins have a wide application prospect in the fields of neuroscience development and neuroscience research and play different physiological functions and therapeutic potentials. Accurate identification of conotoxin types will provide vital clues in revealing the physiological mechanism and pharmacological therapeutic of conotoxins. It is necessary to develop computational tools for both basic research and drug development, particularly for in-depth investigation into the mechanisms of conotoxins and the development of new drugs to treat chronic pain, epilepsy, spasticity, and cardiovascular diseases.

Similarly, the computational-based methods can be also applied to other disulfide-rich venom peptides that target the same ion channels and receptors as conotoxins and show similar pharmacological, biochemical and structural properties, such as spider venoms, centipede or snake venoms.

Although encouraging results have been obtained in conotoxin superfamily and ion channel-target type prediction, further improvements should be made. At first, the prediction accuracy should be further improved. Different types of PseAAC can be applied in the field to formulate conotoxin samples for improving the accuracy. A PSSM (position-specific scoring matrix) produced by similarity search can also be used as an important feature in prediction. Moreover, different feature selection techniques, such as minimal-redundancy-maximal-relevance (mRMR) and principal component analysis (PCA), can also be used to reduce the feature dimension and extract key features. Furthermore, with machine learning approaches, more methods such as deep learning, deep forest, and random forest can also be used to obtain higher accuracies. In addition to superfamily and target type prediction, the signal peptide cleavage sites, the position of two disulfide bonds, and the transition from *L* to *D*-residues are also required to be computationally identified by using machine learning methods. We hope that more and more scholars devote themselves to this field.

## Figures and Tables

**Table 1:** The benchmark datasets of conotoxin superfamily and ion channel-targeted conotoxin.

	**Superfamily**				**Total Number**	**Reference**
	**A**	**M**	**O**	**T**		
S1	25	13	16	17	116	[24,32,34,35]
S2	63	48	95	55	216	[33,36]
	**Type of Ion Channel**				**Total Number**	**Reference**
	**K-Conotoxin**	**Na-Conotoxin**	**Ca-Conotoxin**			
I1	24	43	45		112	[41,42,44,45]
I2	26	49	70		145	[43]

**Table 2:** A list of published results for conotoxin superfamilies and ion channel-targeted conotoxin classifications.

**Superfamily Prediction**								**Reference**
**Dataset**	**Methods**	**A**	**M**	**O**	**T**	**AA**	**OA**	
S1	Multi-class SVMs	0.840	0.923	0.869	0.941	0.893	0.881	[24]
	IDQD	0.960	0.923	0.820	0.940	0.911	0.883	[32]
	SVM-Freescore	0.960	0.984	0.984	1	0.982	0.974	[34]
	Toxin-AAM	0.957	0.966	0.891	0.966	0.945	0.966	[35]
S2	PredCFS	0.960	0.984	0.984	1	0.982	0.903	[33]
	dHKNN	0.957	0.966	0.891	0.966	0.945	0.919	[36]
**Type of Ion Channel-Targeted Prediction**								**Reference**
**Dataset**	**Methods**	**K-Conotoxin**	**Na-Conotoxin**		**Ca-Conotoxin**	**AA**	**OA**	
I1	RBF network	0.917	0.884		0.889	0.897	0.893	[41]
	iCTX-Type	0.833	0.978		0.898	0.903	0.911	[42]
	Fscore-SVM	0.917	0.953		0.953	0.942	0.946	[44]
	AVC-SVM	0.931	0.942		0.892	0.922	0.920	[45]
I2	ICTCPred	1	0.919		1	0.973	0.957	[43]

**Table 3:** A list of the published prediction tools for conotoxin classification.

Name	Prediction Type	URL	Reference
PredCSF	Superfamily	http://www.csbio.sjtu.edu.cn/bioinf/PredCSF/	[33]
ConoDictor	Superfamily	http://conco.ebc.ee	[38]
iCTX-Type	ion channel-target	http://lin.uestc.edu.cn/server/iCTX-Type	[42]
